# Methodology for the inference of gene function from phenotype data

**DOI:** 10.1186/s12859-014-0405-z

**Published:** 2014-12-12

**Authors:** Joao A Ascensao, Mary E Dolan, David P Hill, Judith A Blake

**Affiliations:** The Jackson Laboratory, 600 Main Street, Bar Harbor, ME USA; Rice University, 6100 Main Street, Houston, TX USA

**Keywords:** Gene ontology, Mammalian phenotype ontology, Function prediction, Ontology development

## Abstract

**Background:**

Biomedical ontologies are increasingly instrumental in the advancement of biological research primarily through their use to efficiently consolidate large amounts of data into structured, accessible sets. However, ontology development and usage can be hampered by the segregation of knowledge by domain that occurs due to independent development and use of the ontologies. The ability to infer data associated with one ontology to data associated with another ontology would prove useful in expanding information content and scope. We here focus on relating two ontologies: the Gene Ontology (GO), which encodes canonical gene function, and the Mammalian Phenotype Ontology (MP), which describes non-canonical phenotypes, using statistical methods to suggest GO functional annotations from existing MP phenotype annotations. This work is in contrast to previous studies that have focused on inferring gene function from phenotype primarily through lexical or semantic similarity measures.

**Results:**

We have designed and tested a set of algorithms that represents a novel methodology to define rules for predicting gene function by examining the emergent structure and relationships between the gene functions and phenotypes rather than inspecting the terms semantically. The algorithms inspect relationships among multiple phenotype terms to deduce if there are cases where they all arise from a single gene function.

We apply this methodology to data about genes in the laboratory mouse that are formally represented in the Mouse Genome Informatics (MGI) resource. From the data, 7444 rule instances were generated from five generalized rules, resulting in 4818 unique GO functional predictions for 1796 genes.

**Conclusions:**

We show that our method is capable of inferring high-quality functional annotations from curated phenotype data. As well as creating inferred annotations, our method has the potential to allow for the elucidation of unforeseen, biologically significant associations between gene function and phenotypes that would be overlooked by a semantics-based approach. Future work will include the implementation of the described algorithms for a variety of other model organism databases, taking full advantage of the abundance of available high quality curated data.

**Electronic supplementary material:**

The online version of this article (doi:10.1186/s12859-014-0405-z) contains supplementary material, which is available to authorized users.

## Background

A hallmark of modern biomedical research is the generation of increasingly large amounts of scientific data. Biomedical ontologies have the potential to greatly accelerate biomedical research by enhancing our ability to integrate and access these data. A biomedical ontology is a resource that represents a controlled set of terms for entities in a particular biomedical domain and how those terms are related to one another [1]. Biocurators are scientists who review experimental data, primarily as reported in the biomedical literature, to create empirical connections between different aspects of biological data, that is to say, annotations [2]; *.e.g.* biocurators annotate, or tag, biological entities (e.g. proteins, functional RNAs) with ontology terms (capturing all relevant metadata as well). One of the most widely used modern bio-ontologies is the Gene Ontology (GO), a resource that describes canonical gene functions in a computable species-independent manner so that they may be used for statistical analysis of gene sets or for comparative genomic analysis [3,4]. Another bio-ontology is the Mammalian Phenotype Ontology (MP) that provides an independently curated set of terms and relationships describing non-canonical phenotypes, primarily in mouse models, and that is used to query the effects of genetic mutations [5].

Genes and alleles (genetic variants) of genes are annotated respectively for both function and phenotype within Mouse Genome Informatics (MGI) system, a comprehensive resource for genomic research of the laboratory mouse [6]. Within the MGI curation workflow, different subsets of biocurators separately process papers identified for function (GO) and phenotype (MP) curation. As a result, although papers may be selected for curation in regards to both GO and MP, they are not processed simultaneously, leading to short-term temporal discrepancies in overall curation coverage in MGI. Because scientific literature is published much faster than biocurators can read and curate the papers, the development of methods to computationally infer annotations from one source to another would greatly add and enhance curation efficiency [7].

Recent years have seen efforts to complement curated annotation data sets with text mined and association-rule mined predicted annotations. Broadly, text mining approaches use natural language processing methods to alleviate the backlog of papers awaiting curation, while association-rule mining uses curated annotation sets to predict new annotations and to assess the validity of automated annotation methods [8-17]. It is worth noting that several of these same approaches have been used to improve and expand the ontology structure and relationships as well [18-21]. These efforts have been used both to predict additional annotations from curated annotations in the same ontology and to predict across ontologies, as we do here.

The prediction efforts mentioned above may include lexical matching [8], semantic similarity measures [9-11], ontology matching [18,19], and so-called ‘guilt-by-association’ methods [12]; several efforts use a combination of these approaches [13]. Some prediction methods are primarily ontology based and others are annotation, or instance, based. Our method uses an extension of ‘guilt-by-association’ and is annotation based.

Lexical matching methods, including text mining and text clustering, have been used to infer gene function from phenotype and vice versa. Semantic matching is facilitated by the use within the OBO community of equivalence axioms and logical definitions and by curated inter-ontology links. Semantic similarity measures based on ontology structure or information theory between phenotype and GO have been used to predict additional GO annotations. In a departure from semantic similarity approaches, various groups have performed network analyses to align GO terms with protein association networks to predict protein function [14-16].

Other efforts follow a more empirical approach such as instance based ontology matching and other so-called ‘guilt-by-association’ methods: annotation co-occurrence pairs, knowledge-based annotation inference based on, for example, protein-protein interactions or pathway term enrichment.

Our approach is strictly empirical and makes no assumptions about lexical matching, semantics, or ontology structure, except to infer annotations according to the true-path rule. The rationale behind our approach is to make a simplifying assumption that in some cases ‘interesting’ biology could be missed by limiting the analysis to include an alignment of ontology structure or by attempting to compare the ‘meaning’ of phenotype versus GO terms. Using this simplified approach there is no underlying assumption that ‘similar’ areas of the MPO and the GO should correlate. Instead, we examine the feasibility of constructing rules based only on conjunctions and disjunctions of high-quality phenotype annotations made by MGI curators to predict GO annotations. A crucial difference in our approach is that, where most empirical methods group annotations based on gene entities, our analysis is allele-specific, and therefore addresses the potential that a given set of varied phenotypes may be the result of a single underlying genetic perturbation. Additionally, mouse phenotypes can vary widely for different alleles of the same gene on different strain backgrounds. Indeed, this is the reason for the detailed study of spontaneous and targeted mutations in specific strains of the laboratory mouse. Consider, for example, the phenotypes of three alleles of the mouse *Pax3* gene. One spontaneous allele, *Pax3*^*Sp-d*^ manifests phenotypes in many areas: embryogenesis, integument, limbs/digits/tail, mortality/aging, nervous system, pigmentation. This allele is present in the mouse model for the human disease Waardenburg Syndrome, Type 1; WS1 (OMIM:193500) [22,23]. Another targeted allele, *Pax3*^*tm1Mrc*^ manifests a different set of phenotypes: craniofacial, growth/size, mortality/aging, muscle, nervous system, respiratory, skeleton, tumorigenesis, vision/eye. This allele is present in the mouse model for another human disease Rhabdomyosarcoma 2; RMS2 (OMIM:268220) [24]. Yet another targeted allele, *Pax3*^*tm2.1Joe*^ [25], is present in a mouse in which no abnormal phenotype is observed.

In this work, we describe an original method to predict novel GO annotations for genes associated with alleles that have existing MP annotations. We apply our derived set of rules to a set of papers that have been selected for curation for both MP and GO but that have, as yet, been annotated only for MP, but not for GO, within the MGI system. The algorithms draw inspiration from set and graph theory as they attempt to mathematically analyze relationships between GO and MP term(s). The approach used is as follows: First, gather relevant data and align MP and GO terms based on co-curated literature and shared alleles as our training set; Second, analyze the data such that rules can be made to predict a GO term from MP term(s); And finally, Third, apply the rules to a new set of papers/alleles that are currently annotated for MP and selected for but not yet annotated for GO. The goal of the work is to complement and facilitate the work of biocurators.

## Methods

### Consolidation of datasets

Both MP and GO are used in MGI, an open source resource that freely publishes its datasets. We collected all data used in this study from the MGI ftp site (ftp://ftp.informatics.jax.org/pub/reports/index.html, retrieved 10 June 2013). First, the set of literature that was annotated for both GO and MP in MGI was gathered and formatted; this set provided our training data set (3662 publications with both GO and MP annotations to the same allele). Then, the set of literature selected for both MP and GO, but annotated only for MP (63,028 publications) was collected from internal reports used by curators in their workflow; this set provided the test set for our derived inference rules.

### Data alignment and processing

A base set of MP-GO pairs was generated by matching the set of all collected GO terms to the set of all collected MP terms used to annotate the same allele (we use the allele here as a proxy for full genotype) in the same study, knowledge of which is reflected by shared PubMedID (PMID) and alleleID. The GO terms were filtered to select only those terms from the biological process (BP) subontology, and only those GO annotations with evidence as “inferred from a mutant phenotype” (IMP). These selection criteria provide a defined set of papers restricted formally to those tagged for phenotype-based GO annotation for biological process, provided a first level of quality control on the dataset. There were found to be 81,245 MP-GO pairs sharing PMID/alleleID, and 67,424 unique MP-GO pairs, indicating that many of the MP-GO pairs occurred more than once. The MP, GO and the PMID/alleleID data were modeled as a network with PMID/alleleID nodes connecting MP and GO nodes derived from the same study.

All network visualizations were created using Cytoscape v2.8 [26] and all calculations were performed using Numerical Python (Python 2.7.3, NumPy 1.6.2, SciPy 0.10.1, Matplotlib 1.2) [27]. The network was modeled with an adjacency-like matrix. The term “adjacency matrix” is used loosely here, as the matrices presented in this work are quite different than those traditionally used—the matrices used here are rectangular matrices with the columns representing the annotated terms and rows representing PMID/alleleIDs. This approach is necessary since we wish to track individual studies for which both MP and GO annotations have been made. Two matrices were created, one with all unique PMID/alleleIDs (3662 rows) by all unique GO IDs (2472 columns); the other with all unique PMID/alleleIDs by all unique MP IDs (4978 columns). The network was found to be very sparse—that is, the number of all possible edges far exceeded the number of actual edges (graph density of approximately 6e-04).

### Calculation and evaluation of statistical significance

After constructing our networks, we calculated the probability that a connection between an MP and GO node was statistically significant rather than due to chance alone. Some gene function-phenotype connections might be supported by many shared annotations, implying that there is some underlying connection between the gene function and the mutant phenotype, while others might be connected by only a few connections relative to the number other connections and yet also be informative.

From a set of unique integers of size N representing all the PMID/alleleID combinations, the probability that a selection of n at random will include j or more ‘successes’ – that is, a PMID/alleleID that is shared between a particular MP-GO node pair, can be defined as a modification of the cumulative binomial distribution (for values of N large compared to n):

1 $$ \mathrm{p}\left({\mathrm{X}}_{\mathrm{n}}\ge \mathrm{j}\right)=\kern0.5em {\sum}_{\mathrm{i}\kern0.5em =\kern0.5em \mathrm{j}}^{\mathrm{n}}\left({}_{\mathrm{i}}^{\mathrm{n}}\right)\kern0.5em {\left(\frac{1}{\mathrm{N}}\right)}^{\mathrm{i}}\kern0.5em {\left(1\kern0.5em -\kern0.5em \frac{1}{\mathrm{N}}\right)}^{\mathrm{n}\kern0.5em -\kern0.5em \mathrm{i}}. $$

**Figure 1:**
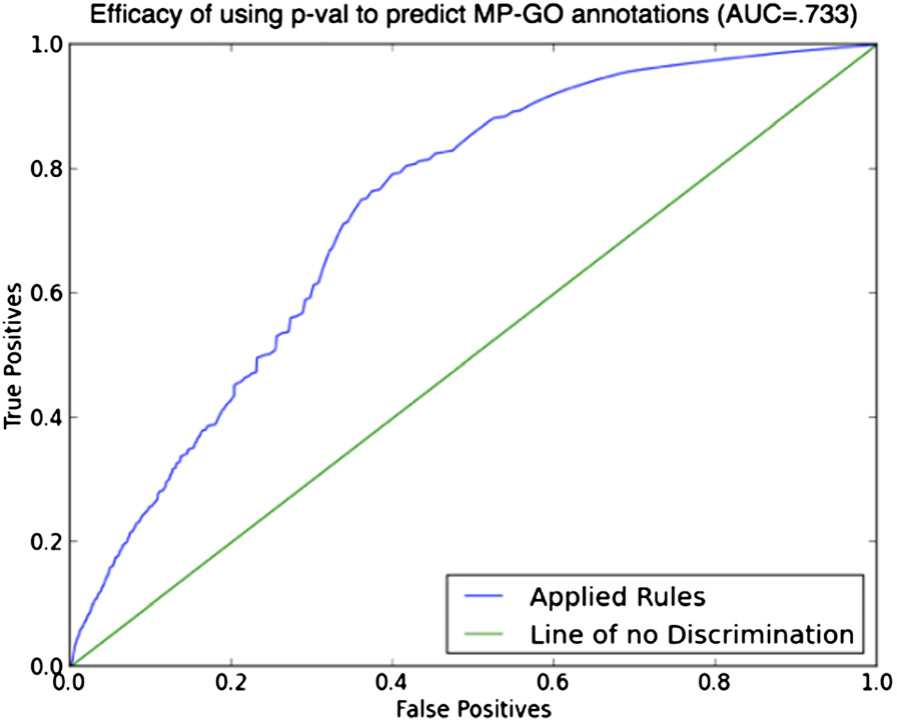
**ROC curve.** Evaluation of the efficacy of a rule: if MP_i_ then GO_j_, using the receiver operating characteristic.

In our case*,* N = 3662, and n, the maximum number of PMID/alleleID combinations between any MP-GO node pair, is 250, so the condition of equation 1 is satisfied. Applying this definition, the total probability, p_tot_, that a particular MP_i_-GO_j_ association is due to chance is given by:

2 $$ {\mathrm{p}}_{\mathrm{tot}} = \mathrm{p}\left[\mathrm{S}\kern0.5em \subseteq \kern0.5em \mathrm{PM}\left(\mathrm{M}{\mathrm{P}}_{\mathrm{i}}\right)\right] $$

where PM(MP_i_) or PM(GO_j_) returns the set of all PMID/alleleID nodes that are connected to the MP_i_ or GO_j_ nodes, respectively, and:

3 $$ \mathrm{S}\kern0.5em =\mathrm{PM}\left(\mathrm{M}{\mathrm{P}}_{\mathrm{i}}\right)\cap \mathrm{PM}\left(\mathrm{G}{\mathrm{O}}_{\mathrm{j}}\right) $$

is the set of *data confirmed* PMID/alleleID connections between the MP_i_ and the GO_j_ nodes. The p-value was calculated in this way for each MP-GO pair found in the data and sorted. The efficacy of the p-value was evaluated by using each MP-GO connection as a prediction, or ‘rule’: MP_i_ → GO_j_ —to wit: “if an annotation of a particular allele has been made to the MP term MP_i_ by curation of a particular study then an IMP annotation corresponding to that allele can be made to the GO term GO_j_.” The resulting true and false positives (sensitivity and specificity) were plotted on the receiver operating characteristic (ROC) curve (Figure 1). As shown, the calculated p-value shows sufficient discriminatory power (AUC = 0.733) to discriminate MP-GO pairs that are more likely to correctly predict a GO node from a given MP term. An alternative scenario was also addressed, where possible MP terms were predicted from alleles with GO annotations (but no MP annotations); it was found that this prediction route does not carry as much predictive value (AUC = .686), further validating our approach to predict GO from MP annotations. We therefore used the calculated p-values as our measure of statistical significance for the GO function prediction from the simple rule.

### Generalized patterns lead to extended rules

While the calculation of the p-value is useful in discovering which MP-GO pairs have stronger connections when run against the training sets, we found that even those with the lowest probabilities of being due to chance returned many false positives. Therefore, the need arose to either better identify the true positives of an MP-GO pair, or to better exclude the false positives: that is, to improve the discriminatory power of our rules.

**Figure 2:**
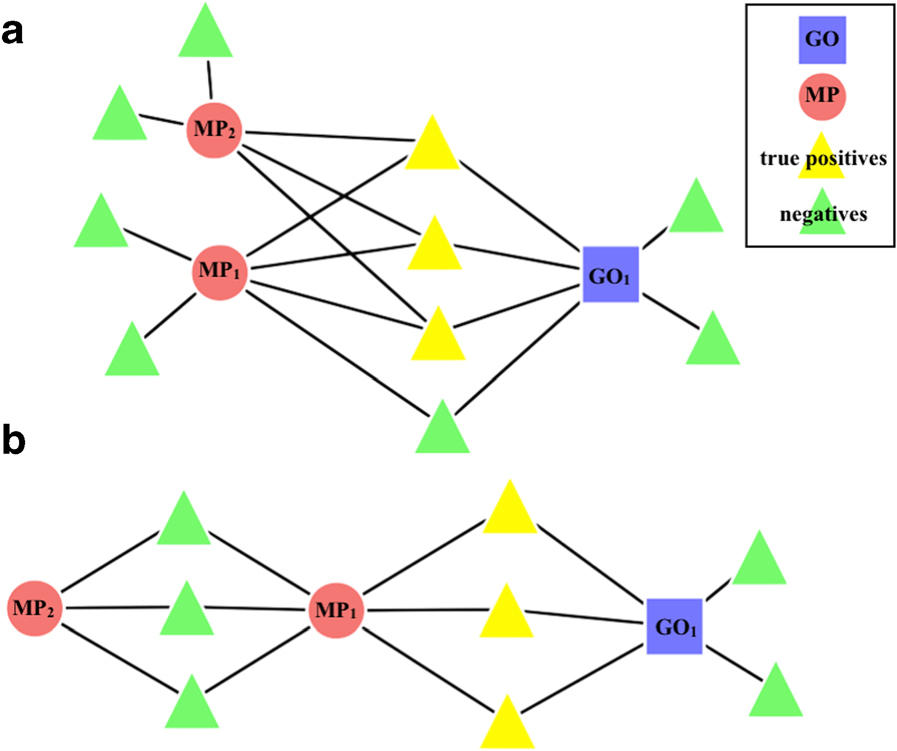
**A graphical representation of the plus and minus rule.** The highlighted nodes represent the ‘successes’ identified by the corresponding rule. **a**, The plus rule-a pattern whereby if MP_1_ and MP_2_, then GO_1_. **b**, The minus rule-another pattern whereby if MP_1_ and not MP_2_, then GO_1_.

Two types of generalized patterns arose from the network, which we chose to identify as the + (plus) rule and the - (minus) rule. The plus-rule can be qualitatively described as inspecting the connections of an MP-GO pair and examining if there is another MP node that is connected to the true positives of the pair, but excludes all of the false positives (PMID/alleleID nodes that are connected to MP, but not to GO) (Figure 2a). We can define the plus-rule: MP_1_ AND MP_2_ → GO_1_ —to wit: “if an annotation of a particular allele has been made to the MP term MP_1_ and to the MP term MP_2_ by curation of a single particular study then an IMP annotation corresponding to that allele can be made to the GO term GO_1_.” The conditional statement for finding an MP_1_ and MP_2_ that follow the pattern of the plus-rule is as follows:

4 $$ \mathrm{PM}\left(\mathrm{M}{\mathrm{P}}_1\right)\cap \mathrm{PM}\left(\mathrm{M}{\mathrm{P}}_2\right)\cap \mathrm{PM}\left(\mathrm{G}{\mathrm{O}}_1\right)==\mathrm{PM}\left(\mathrm{M}{\mathrm{P}}_1\right)\cap \mathrm{PM}\left(\mathrm{M}{\mathrm{P}}_2\right) $$

The minus-rule takes the somewhat opposite approach from the plus-rule; that is, instead of discerning if the intersection of two MP nodes can define the successes, the minus-rule examines if the set *difference* of two MP nodes can define the successes (Figure 2b). Similarly, we can define the minus-rule: MP_1_ AND NOT MP_2_ → GO_1_ —to wit: “if an annotation of a particular allele has been made to the MP term MP_1_ but not to the MP term MP_2_ by curation of a particular study then an IMP annotation corresponding to that allele can be made to the GO term GO_1_.” The statement used to find an MP_1_ and MP_2_ that follow the pattern of the minus-rule is as follows:

5 $$ \left(\mathrm{PM}\left(\mathrm{M}{\mathrm{P}}_1\right)\cap \mathrm{PM}\left(\mathrm{G}{\mathrm{O}}_1\right)\right)\backslash \mathrm{PM}\left(\mathrm{M}{\mathrm{P}}_2\right) = = \mathrm{PM}\left(\mathrm{M}{\mathrm{P}}_1\right)\backslash \mathrm{PM}\left(\mathrm{M}{\mathrm{P}}_2\right) $$

**Figure 3:**
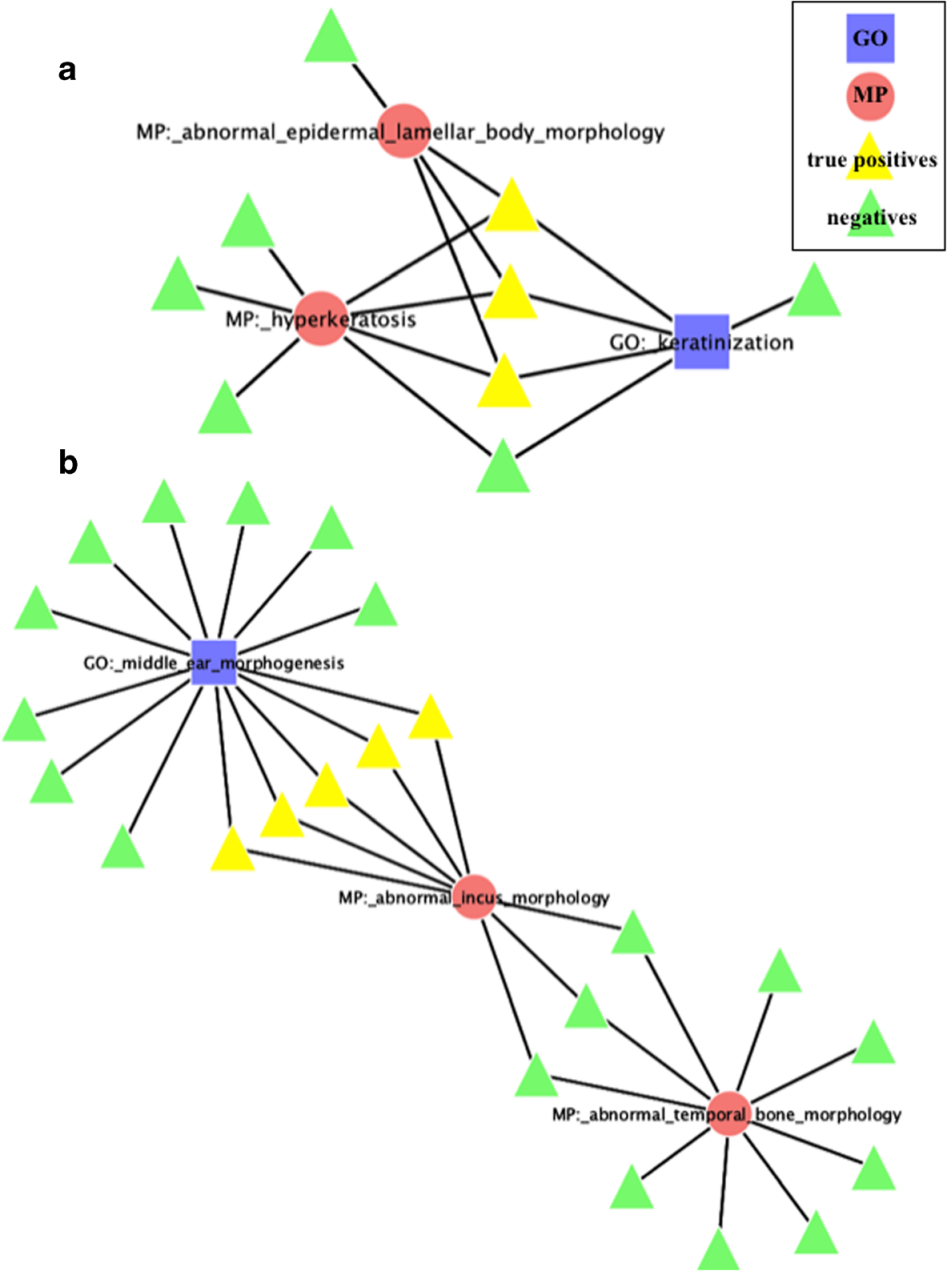
**Example of rule instances.** The highlighted nodes represent the ‘successes’ identified by the corresponding rule. **a**, Plus rule instance. **b**, Minus rule instance.

**Figure 4:**
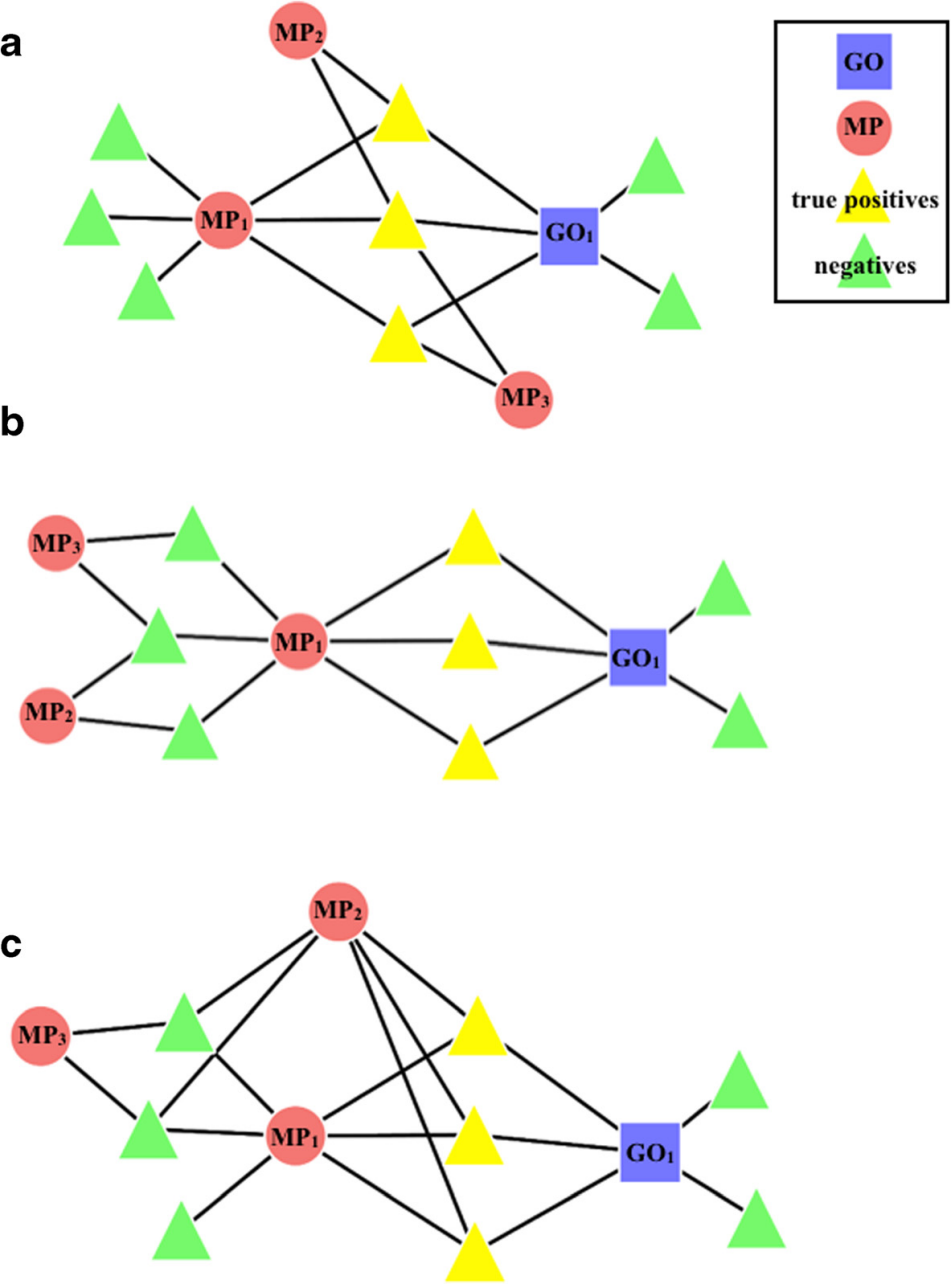
**A graphical representation of the three rules emerging from the plus and minus rules.** The highlighted nodes represent the ‘successes’ identified by the corresponding rule. **a**, The plus-plus rule-if MP_1_ and MP_2_ or MP_3_, then GO_1_. **b**, The minus-minus rule-if MP_1_ and not either MP_2_ or MP_3_, then GO_1_. **c**, The plus-minus rule-if MP_1_ and MP_2_ and not MP_3_, then GO_1_.

The network was then searched for collections of MP and GO nodes that followed the aforementioned patterns, with 3105 instances of the plus-rule pattern and 234 instances of the minus-rule pattern. Detailed examples of both rules are illustrated in Figure 3. However, the need to differentiate between those rules that were more likely to give results to be due to chance versus those that were less likely was still present. Utilizing the demonstrated efficacy of the cumulative binomial distribution as applied to the undirected graph (equation 1), the p-values for the plus and minus-rules were calculated respectively:

6 $$ {\mathrm{S}}_{+}\kern0.5em  = \mathrm{PM}\left(\mathrm{M}{\mathrm{P}}_1\right)\cap \mathrm{PM}\left(\mathrm{M}{\mathrm{P}}_2\right) $$

7 $$ {\mathrm{p}}_{+}\kern0.5em =\kern0.5em \mathrm{p}\left[{\mathrm{S}}_{+}\subseteq \mathrm{PM}\left(\mathrm{M}{\mathrm{P}}_1\right)\right]\ *\ \mathrm{p}\left[{\mathrm{S}}_{+}\subseteq \mathrm{PM}\left(\mathrm{M}{\mathrm{P}}_2\right)\right]*\ \mathrm{p}\left[{\mathrm{S}}_{+}\subseteq \mathrm{PM}\left(\mathrm{G}{\mathrm{O}}_1\right)\right] $$

8 $$ {\mathrm{S}}_{\hbox{-}}\kern0.5em =\kern0.5em \mathrm{PM}\left(\mathrm{M}{\mathrm{P}}_1\right)\backslash \mathrm{PM}\left(\mathrm{M}{\mathrm{P}}_2\right) $$

9 $$ {\mathrm{p}}_{\hbox{-} } = \mathrm{p}\left[{\mathrm{S}}_{\hbox{-}}\subseteq \mathrm{PM}\left(\mathrm{M}{\mathrm{P}}_1\right)\right]\ *\ \mathrm{p}\left[{\mathrm{S}}_{\hbox{-}}\subseteq \mathrm{PM}\left(\mathrm{G}{\mathrm{O}}_1\right)\right]\ *\ \mathrm{p}\left[{{\mathrm{S}}_{\hbox{-}}}^{\mathrm{c}}\subseteq \mathrm{PM}\left(\mathrm{M}{\mathrm{P}}_2\right)\right] $$

where S in each case is the set of ‘successes’ and S^c^ is the set complement. As the network is sparse, the p-values are expected to be small. Arising from the basic plus and minus-rule patterns, three other rule patterns were defined, descriptively designated as plus-plus (++) (Figure 4a), minus-minus (--) (Figure 4b) and plus-minus (+-) (Figure 4c). The mathematical statements used to find rules from three additional rule patterns respectively are as follows:

10 $$ \mathrm{PM}\left(\mathrm{GO}\right)\cap \mathrm{PM}\left(\mathrm{M}{\mathrm{P}}_1\right)\cap \left(\mathrm{PM}\left(\mathrm{M}{\mathrm{P}}_2\right)\cup \mathrm{PM}\left(\mathrm{M}{\mathrm{P}}_3\right)\right)= = \mathrm{PM}\left(\mathrm{M}{\mathrm{P}}_1\right)\cap \left(\mathrm{PM}\left(\mathrm{M}{\mathrm{P}}_2\right)\cup \mathrm{PM}\left(\mathrm{M}{\mathrm{P}}_3\right)\right) $$

11 $$ \mathrm{PM}\left(\mathrm{GO}\right)\cap \mathrm{PM}\left(\mathrm{M}{\mathrm{P}}_1\right)\backslash \left(\mathrm{PM}\left(\mathrm{M}{\mathrm{P}}_2\right)\cup \mathrm{PM}\left(\mathrm{M}{\mathrm{P}}_3\right)\right)= = \mathrm{PM}\left(\mathrm{M}{\mathrm{P}}_1\right)\backslash \left(\mathrm{PM}\left(\mathrm{M}{\mathrm{P}}_2\right)\cup \mathrm{PM}\left(\mathrm{M}{\mathrm{P}}_3\right)\right) $$

12 $$ \left(\mathrm{PM}\left(\mathrm{M}{\mathrm{P}}_1\right)\cap \mathrm{PM}\left(\mathrm{M}{\mathrm{P}}_2\right)\cap \mathrm{PM}\left(\mathrm{GO}\right)\right)\backslash \mathrm{PM}\left(\mathrm{M}{\mathrm{P}}_3\right)= = \left(\mathrm{PM}\left(\mathrm{M}{\mathrm{P}}_1\right)\cap \mathrm{PM}\left(\mathrm{M}{\mathrm{P}}_2\right)\right)\backslash \mathrm{PM}\left(\mathrm{M}{\mathrm{P}}_3\right) $$

The statements used to calculate the p-values for these rule patterns are expounded in the appendix. The network was again searched for arrangements of nodes that followed the three other patterns, with 3143, 328, and 634 instances of the plus-plus-rule, minus-minus-rule and plus-minus-rule respectively. While the composite plus- and minus-rules can be combined iteratively in theory, there seems to be a point where the statements used to calculate the probabilities for large combinations of nodes become too large to be feasibly evaluated, and where they have the potential of straying further from biological reality. Therefore, we limited our analysis to these five rules as examples. All rule instances are compiled in Additional file 1.

### Evaluation of extended rules

All rules were applied to the literature set of PMID/alleleIDs that are annotated to MP term(s) but not yet annotated to a GO term to predict which GO term would be annotated to the PMID/alleleID (set may be found in Additional file 2). Validation of predictions was performed by the selection of a set of 20 papers for each rule that represented a range of the p-values. The papers were read by a GO scientific curator and the curatorial predictions for functional GO annotation were examined in the context of our rule structures. Companion software for the prediction of GO annotations is provided as Additional file 3.

## Results and discussion

### Evaluation of predicted rules

Many of the rules and annotation predictions are immediately intuitive in nature. Table 1 shows a selection of rules of each type (Plus, Minus, …) along with p-values and a preliminary assessment of whether the rule is biologically ‘obvious’ (O) or ‘subtle/surprising’ (S). The inclusion of depth of GO terms as a proxy for specificity provided no additional information. The average depths for various rule sets are: ‘plus’ 7.25; ‘minus’ 7.28; ‘plus-plus’ 7.30; ‘plus-minus’ 7.15; ‘minus-minus’ 7.60. The reviewed annotations showed no trend according to GO depth.

**Table 1:** **Selection of proposed rules**

							**Assessment**
**Rule type**	**Rule number**	**Phenotype terms included or excluded in rule**			**Implied GO term**	**p-value**	**O = obvious**
**Plus-rule**		**MP**_**1**_ **+**	**MP**_**2**_ **+**		**GO**		**S = subtle**
Plus-rule	0	impaired ovarian folliculogenesis	absent mature ovarian follicles		ovarian follicle development	1.47E-36	O
Plus-rule	5	absent Peyer's patches	abnormal spleen morphology		lymph node development	6.82E-30	S
Plus-rule	21	abnormal direction of heart looping	situs inversus		determination of left/right symmetry	1.53E-23	O
**Minus-rule**		**MP**_**1**_ **+**	**MP**_**2**_ **-**		**GO**		
Minus-rule	5	abnormal sperm flagellum morphology	asthenozoospermia		fertilization	2.70E-26	S
Minus-rule	6	abnormal incus morphology	abnormal temporal bone morphology		middle ear morphogenesis	1.45E-25	O
Minus-rule	227	failure of vascular branching	embryonic growth retardation		regulation of angiogenesis	2.49E-10	S
**Plus-Plus-rule**		**MP**_**1**_ **+**	**MP**_**2**_ **+**	**MP** **3** **+**	**GO**		
Plus-Plus-rule	109	increased kidney apoptosis	dilated renal tubules	increased kidney cell proliferation	negative regulation of apoptotic process	1.83E-20	O
Plus-Plus-rule	1188	enlarged liver	hepatic necrosis	increased glycogen level	glycogen metabolic process	1.95E-14	S
**Plus Minus-rule**		**MP**_**1**_ **+**	**MP**_**2**_ **+**	**MP** **3** **-**	**GO**		
Plus Minus-rule	0	globozoospermia	male infertility	absent acrosome	spermatogenesis	4.21E-39	O
Plus Minus-rule	309	abnormal sperm principal piece morphology	male infertility	abnormal sperm axoneme morphology	spermatid development	5.38E-16	O
**Minus-Minus-rule**		**MP**_**1**_ **+**	**MP**_**2**_ **-**	**MP** **3** **-**	**GO**		
Minus-Minus-rule	0	absent mature ovarian follicles	postnatal growth retardation	abnormal female reproductive system morphology	ovarian follicle development	1.10E-31	O
Minus-Minus-rule	8	abnormal lysosome morphology	decreased embryo size	abnormal neuron morphology	lysosome organization	1.91E-29	O

For example, the first Plus-rule #0: If a study shows that an allele has both phenotypes “impaired ovarian folliculogenesis” [MP:0001129] and “absent mature ovarian follicles” [MP:0001132], that allele is predicted be curated to function annotation “ovarian follicle development” [GO:0001541]. This rule has a very low p-value of 1.47E-36 and is assessed as ‘obvious.’ As another example, consider Plus-rule #21: If a PMID/alleleID has both the MP terms “abnormal direction of heart looping” [MP:0004252] and “situs inversus” [MP:0002766], then it should be annotated to the GO term “determination of left/right symmetry” [GO:0007368]. Both phenotypes are *almost always* associated with a defect in the biological process of “determination of left/right symmetry”, and it makes sense that the occurrence of both phenotypes simultaneously would more strongly indicate a defect in the aforementioned process.

Perhaps the more interesting results of our analysis are in the identification of plus-rule statements that are not necessarily obvious, but make sense biologically. For example, Plus-rule #5: If a PMID/alleleID has both the MP terms “absent Peyer’s patches” [MP:0002831] and “abnormal spleen morphology” [MP:0000689] that allele is predicted to have function “lymph node development” [GO:0048535] and is assessed as ‘subtle.’ Many of these rule statements predict relationships that would not result from a purely semantic approach. Plus-plus-rule #1188 predicts that if a PMID/alleleID has the MP terms “enlarged liver” [MP:0000599] and either “hepatic necrosis” [MP:0001654] or “increased glycogen levels” [MP:0005440], then the allele should be annotated to the GO term “glycogen metabolic process” [GO:0005977]. This is interesting because while an “enlarged liver” may not be intuitively or semantically linked to “glycogen metabolic processes”, the liver is instrumental in the storing and metabolism of glycogen, reflecting the overall physiology. This result illustrates another potential use of our correlative analysis in that a perturbation of glycogen metabolism might result in an enlarged liver phenotype or that in an animal with an enlarged liver, but no other phenotype reported, the underlying defect might be in glycogen metabolism. If our method took into consideration the structure of the ontology or a semantic link between terms, the liver correlation would likely have been missed because there is nothing in the ontology itself that states glycogen metabolism and the liver are linked.

### Evaluation of predicted annotations

The next step in our prediction pipeline is the application of our rules to particular allele instances. As a result of this work, 4818 unique potential annotations associated with 1796 genes have been predicted.

Plus-rule #21, discussed above, is predicted to apply to mouse gene *Zic3* [MGI:106676] based on the paper titled “Zic3 is required in the extracardiac perinodal region of the lateral plate mesoderm for left-right patterning and heart development” [PMID:23184148]. The study describes a variety of congenital defects primarily due to defects in the development of embryonic left-right patterning in *Zic3*^*tm1Bca*^ mice [28]. Our prediction is that since the paper has been used to make annotations to the MP terms “abnormal direction of heart looping” [MP:0004252] and “situs inversus” [MP:0002766], then it would be annotated, or should be annotated, to the GO term “determination of left/right symmetry” [GO:0007368]. In this case, curatorial review confirmed that the rule held true.

Tables 2, 3, 4, 5, and 6 show the curator’s evaluation of the annotations derived from the Plus, Minus, Plus-plus, Minus-minus, Plus-minus-rules respectively for the 20 papers that were reviewed for each rule type.

**Table 2:** **Validation of annotations derived from plus-rules**

**Rule number**	**Gene**	**Phenotype terms included in rule**		**Implied GO term**		
		**MP**_**1**_ **+**	**MP**_**2**_ **+**	**GO**	**p-value**	**Valid?**
0	Foxl2	ovarian follicle development	impaired ovarian folliculogenesis	absent mature ovarian follicles	1.47E-36	yes
1	Mbd4	determination of left/right symmetry	abnormal left-right axis patterning	situs inversus	6.13E-36	yes
2	Ar	spermatogenesis	small testis	decreased testis weight	3.47E-33	yes
2	Ar	spermatogenesis	small testis	decreased testis weight	3.47E-33	yes
2	Ar	spermatogenesis	small testis	decreased testis weight	3.47E-33	yes
2	Hsp90aa1	spermatogenesis	small testis	decreased testis weight	3.47E-33	yes
3	Psip1	anterior/posterior pattern specification	thoracic vertebral transformation	abnormal rib morphology	3.76E-33	yes
4	Fen1	lung development	pulmonary hypoplasia	decreased lung weight	2.48E-30	yes
5	Rag2	lymph node development	absent Peyer's patches	abnormal spleen morphology	6.82E-30	yes
6	Tapt1	anterior/posterior pattern specification	thoracic vertebral transformation	abnormal rib-sternum attachment	3.01E-29	yes
3072	Mir140	decreased body size	decreased length of long bones	collagen fibril organization	2.36E-08	no
3087	Fancm	premature death	abnormal ovary morphology	cell proliferation	4.05E-08	yes
3091	Flnb	abnormal angiogenesis	decreased body weight	angiogenesis	5.29E-08	yes
3096	Slc6a8	abnormal spatial learning	decreased body weight	learning or memory	6.56E-08	yes
3098	Tgfb1	abnormal extraembryonic tissue morphology	decreased body size	gastrulation with mouth forming second	8.17E-08	no
3099	Sirt1	decreased embryo size	ventricular septal defect	neural tube closure	8.29E-08	yes
3100	Kcnq1	deafness	decreased body weight	neuromuscular process controlling balance	1.06E-07	yes
3100	Slc12a7	deafness	decreased body weight	neuromuscular process controlling balance	1.06E-07	no
3101	Sall4	open neural tube	premature death	neural tube closure	1.09E-07	yes
3103	Pkhd1	respiratory failure	postnatal growth retardation	lung development	1.31E-07	no

**Table 3:** **Validation of annotations derived from minus-rules**

**Rule number**	**Gene**	**Phenotype Terms included in rule**		**Implied GO term**		
		**MP**_**1**_ **+**	**MP**_**2**_ **-**	**GO**	**p-value**	**Valid?**
0	Fn1	abnormal vitelline vascular remodeling	complete embryonic lethality during organogenesis	angiogenesis	2.86E-41	yes
1	Gja1	absent mature ovarian follicles	postnatal growth retardation	ovarian follicle development	8.95E-32	yes
1	Stra8	absent mature ovarian follicles	postnatal growth retardation	ovarian follicle development	8.95E-32	yes
2	Ppp1cc	globozoospermia	absent acrosome	spermatogenesis	8.65E-28	yes
4	Tert	thoracic vertebral transformation	cervical vertebral transformation	anterior/posterior pattern specification	2.45E-26	yes
4	Kat2a	thoracic vertebral transformation	cervical vertebral transformation	anterior/posterior pattern specification	2.45E-26	yes
5	Krt19	abnormal sperm flagellum morphology	asthenozoospermia	fertilization	2.70E-26	no
5	Akap4	abnormal sperm flagellum morphology	asthenozoospermia	fertilization	2.70E-26	no
6	Emx2	abnormal incus morphology	abnormal temporal bone morphology	middle ear morphogenesis	1.45E-25	yes
7	Lmx1a	head tossing	circling	auditory receptor cell stereocilium organization	1.02E-24	no
8	Bmp7	palatal shelves fail to meet at midline	cleft secondary palate	thyroid gland development	1.37E-23	no
214	Sctr	decreased urine sodium level	decreased body weight	negative regulation of blood pressure	3.25E-11	no
217	Fbn1	abnormal intercostal muscle morphology	respiratory failure	somitogenesis	5.27E-11	no
218	Krt19	short sperm flagellum	asthenozoospermia	cilium morphogenesis	5.46E-11	yes
220	Rab38	abnormal pulmonary acinus morphology	respiratory distress	lung development	5.84E-11	no
224	Ube2b	abnormal double-strand DNA break repair	small testis	synapsis	1.33E-10	yes
227	Adam10	failure of vascular branching	embryonic growth retardation	regulation of angiogenesis	2.49E-10	no
229	Casr	decreased circulating calcium level	postnatal growth retardation	calcium ion transport	4.98E-10	no
230	Hes7	abnormal sacral vertebrae morphology	complete neonatal lethality	embryonic skeletal system development	5.26E-10	yes
231	Bhlhe22	abnormal corticospinal tract morphology	complete neonatal lethality	adult walking behavior	5.26E-10	no

**Table 4:** **Validation of annotations derived from plus-plus-rules**

**Rule number**	**Gene**	**Phenotype terms included in rule**			**Implied GO term**		
		**MP**_**1**_ **+**	**MP**_**2**_ **+**	**MP** **3** **+**	**GO**	**p-value**	**Valid?**
0	Foxl2	absent mature ovarian follicles	impaired ovarian folliculogenesis	decreased uterus weight	ovarian follicle development	4.15E-37	yes
0	Fads2	absent mature ovarian follicles	impaired ovarian folliculogenesis	decreased uterus weight	ovarian follicle development	4.15E-37	yes
0	Kiss1	absent mature ovarian follicles	impaired ovarian folliculogenesis	decreased uterus weight	ovarian follicle development	4.15E-37	yes
6	Mapk6	decreased lung weight	small lung	pulmonary hypoplasia	lung development	9.35E-31	yes
17	Kiss1r	decreased uterus weight	decreased ovary weight	decreased uterus weight	ovarian follicle development	1.11E-27	yes
17	Esr1	decreased uterus weight	decreased ovary weight	decreased uterus weight	ovarian follicle development	1.11E-27	no
22	Ndrg2	sacral vertebral transformation	lumbar vertebral transformation	sacral vertebral transformation	anterior/posterior pattern specification	3.92E-27	yes
27	Gmcl1	abnormal acrosome morphology	abnormal sperm flagellum morphology	arrest of spermiogenesis	acrosome assembly	2.48E-24	yes
28	Gdf6	abnormal middle ear ossicle morphology	abnormal middle ear morphology	abnormal stapes morphology	middle ear morphogenesis	7.47E-24	yes
48	Gucy2d	abnormal olfactory system physiology	abnormal odor adaptation	abnormal olfactory system physiology	sensory perception of smell	4.15E-22	yes
83	Tnfrsf11b	abnormal middle ear ossicle morphology	abnormal middle ear morphology	abnormal incus morphology	middle ear morphogenesis	6.33E-21	yes
109	Bag6	increased kidney apoptosis	dilated renal tubules	increased kidney cell proliferation	negative regulation of apoptotic process	1.83E-20	yes
150	Mns1	kinked sperm flagellum	oligozoospermia	hairpin sperm flagellum	spermatid development	4.96E-20	yes
719	Pou1f1	abnormal cerebellar foliation	tremors	abnormal cerebellar granule layer	neuron migration	5.59E-16	yes
763	Prdm9	abnormal double-strand DNA break repair	abnormal ovary morphology	decreased oocyte number	double-strand break repair	8.50E-16	yes
812	Camk4	increased bone mineral density	decreased osteoclast cell number	failure of tooth eruption	ossification	1.27E-15	no
814	Ddr2	decreased circulating testosterone level	absent corpus luteum	Leydig cell hypoplasia	spermatogenesis	1.31E-15	yes
816	Bmper	curly tail	short tail	abnormal rib-vertebral column attachment	skeletal system morphogenesis	1.33E-15	yes
1373	Sec61a1	enlarged liver	increased circulating triglyceride level	increased glycogen level	glycogen metabolic process	6.61E-14	no
1709	Ctsc	decreased circulating interleukin-6 level	decreased susceptibility to endotoxin shock	decreased circulating interleukin-6 level	response to lipopolysaccharide	4.38E-13	no

**Table 5:** **Validation of annotations derived from plus-minus-rules**

**Rule number**	**Gene**	**Phenotype terms included in rule**			**Implied GO term**		
		**MP**_**1**_ **+**	**MP**_**2**_ **+**	**MP** **3** **-**	**GO**	**p-value**	**Valid?**
0	M1ap	globozoospermia	male infertility	absent acrosome	spermatogenesis	4.21E-39	yes
0	Ing2	globozoospermia	male infertility	absent acrosome	spermatogenesis	4.21E-39	yes
1	Cdk16	abnormal sperm flagellum morphology	male infertility	teratozoospermia	cilium morphogenesis	1.71E-28	yes
8	Adipor2	abnormal glucose homeostasis	decreased circulating insulin level	increased insulin sensitivity	positive regulation of insulin secretion	1.49E-24	yes
10	Steap3	polychromatophilia	decreased hemoglobin content	anisocytosis	skeletal system morphogenesis	4.46E-24	no
13	Ptges3	abnormal production of surfactant	atelectasis	cyanosis	lung alveolus development	8.94E-24	yes
19	Mbl1	increased IgG2a level	increased IgG1 level	abnormal T cell physiology	negative regulation of B cell proliferation	5.82E-23	no
47	Kat2a	decreased body size	exencephaly	domed cranium	neural tube closure	3.40E-20	yes
77	Siglec1	decreased tumor necrosis factor secretion	decreased interleukin-6 secretion	abnormal macrophage physiology	cellular response to lipopolysaccharide	2.95E-19	yes
96	Chga	dilated heart left ventricle	heart left ventricle hypertrophy	absent caveolae	heart morphogenesis	1.56E-18	no
118	Mark3	improved glucose tolerance	decreased circulating insulin level	abnormal glucose homeostasis	response to insulin	3.83E-18	no
124	Gpr64	kinked sperm flagellum	teratozoospermia	hairpin sperm flagellum	fertilization	5.57E-18	no
139	Pick1	abnormal acrosome morphology	asthenozoospermia	absent acrosome	spermatogenesis	8.55E-18	yes
153	Col2a1	abnormal long bone metaphysis morphology	abnormal long bone epiphyseal plate proliferative zone	protruding tongue	bone morphogenesis	1.35E-17	yes
167	Odf1	male infertility	impaired acrosome reaction	impaired fertilization	acrosome reaction	1.91E-17	yes
177	Cftr	absent estrous cycle	small ovary	increased follicle stimulating hormone level	spermatogenesis	2.26E-17	no
262	Zfpm1	abnormal common myeloid progenitor cell morphology	extramedullary hematopoiesis	decreased B cell number	T cell differentiation	1.81E-16	no
309	Gpx4	abnormal sperm principal piece morphology	male infertility	abnormal sperm axoneme morphology	spermatid development	5.38E-16	yes
331	Pank1	increased body weight	hepatic steatosis	abnormal abdominal fat pad morphology	lipid metabolic process	7.25E-16	yes
391	Cd36	increased circulating triglyceride level	abnormal glucose homeostasis	insulin resistance	glycogen metabolic process	2.72E-15	no

**Table 6:** **Validation of annotations derived from minus-minus-rules**

**Rule number**	**Gene**	**Phenotype terms included in rule**			**Implied GO term**		
		**MP**_**1**_ **+**	**MP**_**2**_ **+**	**MP** **3** **-**	**GO**	**p-value**	**Valid?**
0	Esr1	absent mature ovarian follicles	postnatal growth retardation	abnormal female reproductive system morphology	ovarian follicle development	1.10E-31	yes
0	Tex12	absent mature ovarian follicles	postnatal growth retardation	abnormal female reproductive system morphology	ovarian follicle development	1.10E-31	no
0	Zglp1	absent mature ovarian follicles	postnatal growth retardation	abnormal female reproductive system morphology	ovarian follicle development	1.10E-31	no
0	Syce3	absent mature ovarian follicles	postnatal growth retardation	abnormal female reproductive system morphology	ovarian follicle development	1.10E-31	no
0	Gja1	absent mature ovarian follicles	postnatal growth retardation	abnormal female reproductive system morphology	ovarian follicle development	1.10E-31	yes
1	Stra8	absent mature ovarian follicles	postnatal growth retardation	decreased compact bone thickness	ovarian follicle development	1.15E-31	yes
8	Idua	abnormal lysosome morphology	decreased embryo size	abnormal neuron morphology	lysosome organization	1.91E-29	yes
8	Sort1	abnormal lysosome morphology	decreased embryo size	abnormal neuron morphology	lysosome organization	1.91E-29	yes
12	Zbtb20	abnormal hippocampus development	complete neonatal lethality	abnormal forebrain development	hippocampus development	5.56E-26	yes
15	Runx1	abnormal embryonic hematopoiesis	complete embryonic lethality during organogenesis	anemia	spongiotrophoblast layer development	2.53E-22	no
15	Pdgfrb	abnormal embryonic hematopoiesis	complete embryonic lethality during organogenesis	anemia	spongiotrophoblast layer development	2.53E-22	no
119	Jak2	polychromatophilia	partial postnatal lethality	increased lactate dehydrogenase level	erythrocyte differentiation	1.15E-11	no
120	Gjb2	abnormal mesenchyme morphology	embryonic growth retardation	internal hemorrhage	neural tube closure	1.82E-11	no
129	T	abnormal limb bud morphology	complete perinatal lethality	abnormal digit morphology	anterior/posterior pattern specification	2.93E-11	yes
140	Stk4	decreased spleen white pulp amount	postnatal growth retardation	arrested B cell differentiation	lymph node development	3.39E-11	no
173	Mapk7	abnormal head mesenchyme morphology	abnormal blood vessel morphology	abnormal heart tube morphology	neural tube closure	6.48E-11	no
188	Rtel1	enlarged allantois	no abnormal phenotype detected	small otic vesicle	gastrulation	1.67E-10	no
212	Smad4	abnormal proximal-distal axis patterning	embryonic growth arrest	failure to gastrulate	proximal/distal pattern formation	1.94E-10	no
219	Adam10	failure of vascular branching	embryonic growth retardation	enlarged pericardium	regulation of angiogenesis	2.53E-10	no
228	Rapgef2	abnormal embryonic hematopoiesis	complete embryonic lethality during organogenesis	abnormal liver morphology	spongiotrophoblast layer development	3.70E-10	no

In 16 out of 20 cases, the Plus-rule generated an annotation that was considered correct by the curator. In all cases, the incorrect inferences were associated with poorer p-values (Table 2), validating its utility as a score to measure confidence level. In one negative case shown here, Plus-rule 3072: “decreased body size” plus “decreased length of long bones” phenotypes implies “collagen fibril organization,” the curator specifically noted that a curator could not make this annotation based on the paper since the authors did not look at the collagen fibrils directly although they did show that the cartilage in the developing bone is affected Another interesting negative case is the application of Plus-rule #3100: “deafness” plus “decreased body weight” phenotypes implies “neuromuscular process controlling balance” to two different genes. For *Kcnq1*, based on the paper [PMID:15498462], the annotation could be made; while for *Slc12a7*, based on the paper [PMID:11976689], it could not.

In the case of the Minus-rule, only 10 of the 20 predicted annotations were correct. In the case of the Minus-rule, better p-value scores gave more reliable predictions (Table 3). The Plus-plus (16 correct of 20) and Minus-minus (7 correct of 20) rules displayed similar behavior to the plus and minus-rules respectively (Tables 4 and 5), while the Plus-minus-rule (12 correct of 20) unsurprisingly seemed at almost act as an intermediate between the Plus and Minus-rules (Table 6).

Testing the validation of the various rules showed that, in general, the Plus-rule statements are more reliable that the minus-rule statements in generating valid annotations. This is not surprising for two reasons: (1) combined phenotypes often give ‘additive’ evidence to support the GO annotation and (2) the ‘closed world’ assumption limits the predictive power of the absence of annotation. The value of ‘additive’ evidence is mentioned above for Plus-rule #0, “absent mature ovarian follicles” [MP:0001132] plus “impaired ovarian folliculogenesis” [MP:0001129] additively support that a gene product would be directly involved in the GO process “ovarian follicle development” [GO:0001541]. The ‘closed world assumption’ is the assumption that what is not currently known to be true is assumed to be false. In our case, the Minus-rules rely on the lack of phenotype annotation as if it the phenotype was tested and found not to obtain.

**Figure 5:**
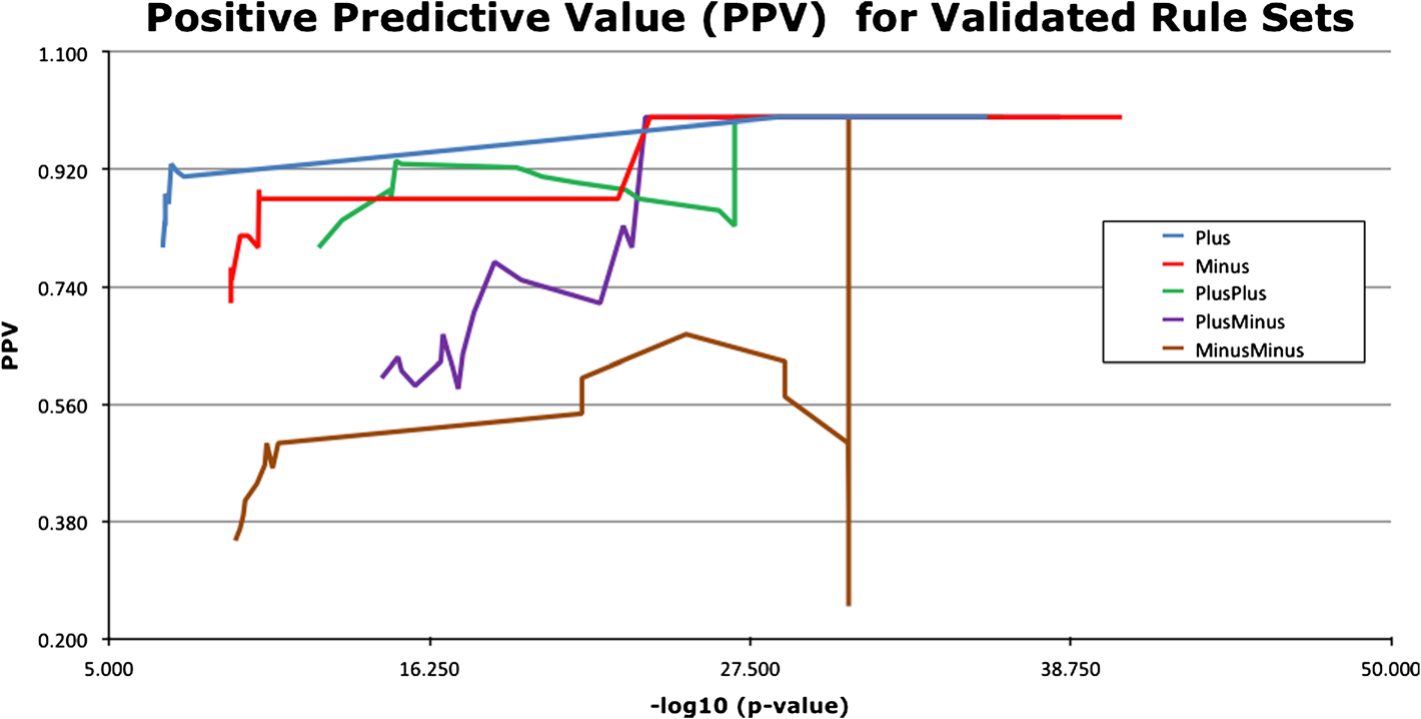
**Comparison of the Positive Predictive Value for various p-value cutoffs of the composite rules for the reviewed annotation predictions.** The PPV for the first three composite rules is markedly better for a given p-value than for the last two rules.

We did a rather simple comparison of the predictive power of our rules based on the reviewed annotation predictions. We calculated the Positive Predictive Value from the true positives (TP) and false positives (FP) in the reviewed annotation sets for each of the composite rules: PPV = TP/(TP + FP). Figure 5 shows a comparison of the PPV for various p-value cutoffs of the composite rules for the reviewed annotation predictions. The PPV for the first three composite rules is markedly better for a given p-value than for the last two rules.

Our analysis shows that the Minus-rule statements are not reliable at predicting specific GO annotations. However, these results are very interesting because although the rules failed to accurately predict correct GO terms, the general area of biology of the predictions was often accurate. In several cases we found that the processes predicted might lead to downstream phenotypic similarities that would fit the phenotypes given in the rules. For example, the terms that predicted the process of ‘fertilization’ (Minus-rule 5) were not entirely correct, we noted, because the gene products identified did affect sperm motility and would create defective sperm, but we could not conclude from the papers [PMID:16015579; PMID:12167408] that the predicted gene products contributed to “fertilization” [GO:0009566] which in GO is specifically defined as the union of the two gametes. Likewise, the prediction of “regulation of angiogenesis” [GO:0045765] would have been correct if the prediction were “vasculogenesis” [GO:0001570], both of which can lead to abnormal branching of blood vessels (Minus-rule 227). These types of predictions may still prove useful for curators in that they point to relevant branches of biology for annotation consideration.

Our results show that the use of correlation modeling can be used to infer biological knowledge about the processes that underlie phenotypic expression. We show that the use of the Plus-rule statements has the potential to accurately predict GO annotations, and that while the Minus-rule generally predicts an incorrect specific term, it often predicts a correct area of biology. Our results show that curators can use our correlative rules to guide manual curation however, individual instances should not necessarily be annotated without the review of a biocurator. The rules can be used to help curators decide about a general subcategorization of GO terms from which to choose when curating GO data based on mutant phenotypes. The method could also be used when biocurators are trying to identify literature that covers an area of biology of interest. As the rules are further tested and more papers are cocurated for phenotype and GO, the rules will become further refined and accurate p-value cutoffs for reliability can be determined.

Future work will use these methods to create and test more complex rules based on this strategy. Our methodology could be used in future work to help predict the biological process that underlies a given disease by correlating the disease-phenotypes with GO biological processes. This methodological approach can be adapted to any species with rich phenotypic data. Lastly, we could similarly reverse the method to use multiple GO terms to predict likely phenotypic outcomes such as the disruption of specific pathways and then test those predictions as potential new mouse models of human disease.

## Conclusions

Our correlative analysis for predicting GO annotations can be used to assist biocurators in the curation of papers with no previous GO annotations while also giving potential insight into complex phenotype-gene function relationships. We believe this is the first attempt to predict a GO term from its composite relationship with MP terms independently of semantic analysis but rather through a shared allele, and to apply that method to predict GO annotations to a set of papers that have not yet been annotated for GO. Our method has the advantage in that since it does not take semantic similarity into account, it can potentially find correlations between phenotypes and biological processes that are not intuitively obvious or that are not explicitly states in either ontology. Of course we are in a good position to perform this type of analysis since there are large independent efforts in the MGI resource to annotate both phenotypes and GO. The independent annotation serves as an internal control in that the annotations that are in the current corpus are essentially ‘blind’ with respect to one another. Co-curation of phenotype and underlying biological processes could potentially change our results dramatically since a single curator could be swayed to search for a semantic similarity in terms chosen from each ontology. One interesting experiment would be to perform this same analysis on a data set from another group in which individual curators annotate to both ontologies. Since our methodology is purely correlative and does not rely on any other metric, it could potentially be used with other data sets such a GO terms and disease ontologies, or even in combination with several ontologies such as GO, phenotype and expression. The five rules analyzed in this work can be combined in various manners to create many more possible derived rule structures—our results here serve as a ‘proof of principal’ for this type of analysis and pave the way for future iterations.

In practice, we have developed a script and methodology that, given a gene/PubMed ID, will suggest a GO term(s) to a curator if it meets the requirements of one of our rule statements. We intend to integrate this immediate approach into the workflow of MGI curators and hope that others will use our methodology to explore correlative algorithms in the context of diverse data sets.

## Additional files

Additional file 1:
**5 files, where [RuleName] is either Plus, Minus, Plus-Plus, Minus-Minus, or Plus-Minus, corresponding to the rules described in this work. **The files include the instances of all the rules and the calculated p-value. Additional file 2:
**5 files, where [RuleName] follows the same pattern as described above.** The files include all of the PMIDs predicted from the rules described, along with the corresponding p-value. Additional file 3:
**Contains a simple companion software with documentation that uses data updated in real time to infer GO annotations given an MGI ID.** Inputs: MGI ID, PMID (optional); Outputs: Rule #, Inferred GO, PMID, p-val.

## Abbreviations

GO: Gene ontology
MP: Mammalian phenotype ontology
MGI: Mouse genome informatics
PMID: PubMed identification number
IMP: Inferred from mutant phenotype
BP: Biological process
