# Supplementary material for: Methodology for the inference of gene function from phenotype data
Source: BMC Bioinformatics. 2014 Dec 12;15(1):405. doi: 10.1186/s12859-014-0405-z (PMC4302099; doi:10.1186/s12859-014-0405-z)

MP2GO User Documentation

Joao A Ascensao, Mary E Dolan, David P Hill, Judith A Blake (2014)

I. Introduction

MP2GO is a scientific computing software designed to aide curators in annotating genes with no Gene Ontology (GO) annotations, but with alleles that have several Mammalian Phenotype Ontology (MP) annotations. Currently, the software has been implemented with mouse (*Mus musculus*) data from Mouse Genome Informatics (MGI), updated in real time.

A gene identifier is entered into the software, which then uses the methodology described in Ascensao et al. to infer GO annotations for the gene.

Python 2.7 must be installed to run. MP2GO may start-up slowly on some machines (~1-2min). However, all subsequent calls typically run at normal speeds.

II. Basic Functions

At start-up, basic instructions will be displayed along with an input for an MGI ID. The user may then input a PubMed ID (PMID) to restrict results from a specified literature source; this field may be left blank if desired. As multiple inferences are often generated, the user may then sort the resulting table by rule number (default if field left blank), GO ID, PMID or p-val.

For example:

```
MGI ID: MGI:95566
PMID (optional): 17336907
Sort by RULE (default), GO, PMID or PVAL?: PVAL
```

III. Exporting Data

After table of inferred GO annotations is generated, user will be asked if they want to save the data. If yes (Y), an interface will open to ask the user to direct the exported to a specified location and name. The default file extension is .csv.

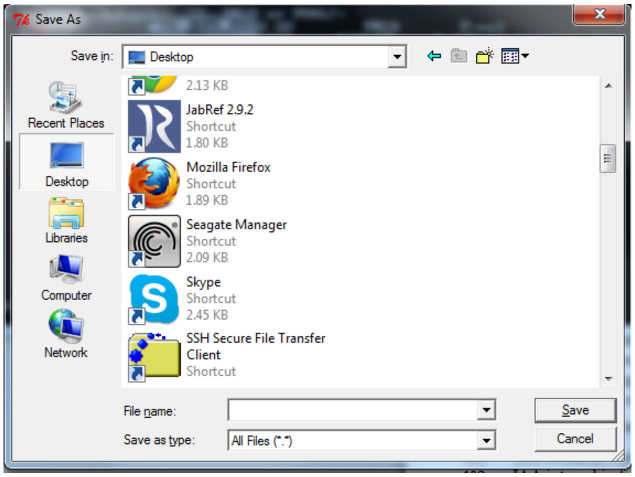

Supplement: Additional file 2: — 5 files, where [RuleName] follows the same pattern as described above. The files include all of the PMIDs predicted from the rules described, along with the corresponding p-value. [file 12859_2014_405_MOESM2_ESM.zip › MP2GO Documentation.pdf]
